# Anti-Aging Effects of *Vaccinium bracteatum* Thunb. Leaves Extracts via Activation of the Nrf2 Antioxidant Pathway

**DOI:** 10.3390/foods15081393

**Published:** 2026-04-16

**Authors:** Caiyun Zhang, Qing Hu, Fenfa Li, Jianming Luo, Liu Liu, Xichun Peng

**Affiliations:** Department of Food Science and Engineering, Jinan University, Guangzhou 510632, China

**Keywords:** *Vaccinium bracteatum* Thunb. leaves, xanthotoxol, antioxidant, anti-aging, Nrf2 pathway

## Abstract

*Vaccinium bracteatum* Thunb. leaves (VBTL), a traditional medicinal plant historically consumed as food in certain regions of China, have been documented to possess potent in vitro antioxidant activity. However, its in vivo anti-aging effects and underlying mechanisms remain to be fully elucidated. Therefore, this study aimed to evaluate its anti-aging efficacy to support its potential value as a functional food constituent for healthy aging. Anti-aging efficacy was systematically assessed using D-galactose-induced aging mice, a *Caenorhabditis elegans* model, and an H_2_O_2_-induced cellular senescence model. Key active constituents were identified via untargeted metabolomics. In D-galactose-induced aging mice, VBTL extracts effectively ameliorated oxidative stress, significantly increasing the activities of endogenous antioxidant enzymes such as superoxide dismutase (SOD) and catalase (CAT), while reducing malondialdehyde (MDA) levels. In *Caenorhabditis elegans*, VBTL extended lifespan, reduced lipofuscin accumulation, and demonstrated no reproductive toxicity. Untargeted metabolomics identified xanthotoxol as a key active constituent, which was then selected for mechanistic investigation. In a cellular senescence model, xanthotoxol alleviated H_2_O_2_-induced oxidative stress, significantly enhanced SOD activity, reduced reactive oxygen species (ROS) and MDA levels, inhibited senescence-associated β-galactosidase (SA-β-gal) activity and the expression of senescence-associated secretory phenotype (SASP) factors (IL-6, MMP1, MMP3), and downregulated the expression of genes in the *P53*/*P21*/*P16* signaling pathway. Mechanistically, xanthotoxol activated the Nrf2 signaling pathway, promoting the expression of its downstream targets heme oxygenase-1 (HO-1) and NAD(P)H quinone oxidoreductase 1 (NQO1). This study demonstrates that VBTL and its active compound xanthotoxol exert anti-aging effects across multiple models by modulating the Nrf2 pathway, providing both theoretical and experimental foundations for developing VBTL as a novel, safe, and effective natural ingredient in anti-aging functional foods.

## 1. Introduction

Aging entails the progressive loss of physiological homeostasis, a functional decline in tissues, and an increased susceptibility to age-related diseases [1]. The underlying mechanisms are multifactorial and involve genetics, environmental factors [2], metabolism [3], and oxidative stress [4]. Among these, oxidative stress is widely recognized as a central mechanism driving the onset and progression of aging. During normal metabolism, organisms continuously generate ROS. Excessive ROS levels induce oxidative damage to lipids, proteins [5], and DNA [6], leading to cellular dysfunction, senescence, or death. To counteract oxidative stress and maintain redox homeostasis, organisms employ an endogenous antioxidant defense system comprising key enzymes such as SOD, CAT, and glutathione peroxidase (GSH-Px) [7]. The nuclear factor erythroid 2-related factor 2 (Nrf2) signaling pathway acts as a master molecular switch regulating this defense system. By upregulating the transcription of downstream antioxidant genes, the Nrf2 pathway exerts a pivotal function in mitigating aging and related diseases [8,9].

The D-gal-induced aging model is a well-established experimental system for evaluating the anti-aging potential of natural products. This model is characterized by behavioral deficits [10], excessive ROS accumulation, reduced antioxidant capacity, and pathological tissue damage. In addition, H_2_O_2_ has been widely employed to induce oxidative stress and cellular aging in vitro. At elevated concentrations, H_2_O_2_ induces oxidative damage via protein carbonylation, lipid peroxidation, and DNA damage, thereby directly driving cellular senescence [11].

**Figure A1:**
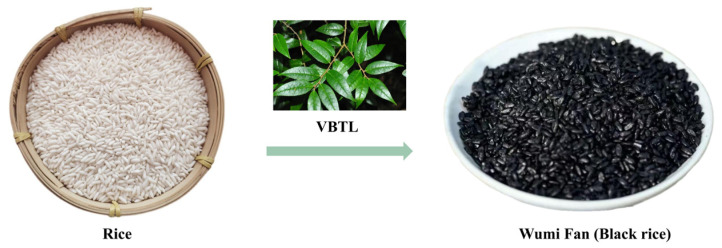
The preparation process of Wumi rice.

Naturally derived bioactive compounds have attracted considerable research interest due to their favorable safety profiles, minimal side effects, and diverse health-promoting activities, with applications extending from pharmacological research to functional foods and nutraceuticals. The exploration of edible plants with a history of traditional consumption holds distinct value, as their long-term safety is often empirically supported, thereby facilitating their translation into dietary strategies for healthy aging. Within the genus *Vaccinium*, existing studies have predominantly focused on the biological activities of fruit extracts. Research has demonstrated that fruit extracts from species such as bilberry (*Vaccinium myrtillus* L.), bog bilberry (*Vaccinium uliginosum* L.), and blueberry (*Vaccinium corymbosum* L.) possess significant health benefits, including antioxidant, anti-inflammatory, and anti-aging effects [12,13,14,15]. In contrast, studies on the leaves of *Vaccinium* species remain relatively limited. *Vaccinium bracteatum* Thunb. (commonly known as Oriental Blueberry or “Wu Fan Shu”) exemplifies a plant with dual utility in traditional medicine and cuisine. While its leaves (VBTL) are documented in classical pharmacopoeias for medicinal purposes, their most distinctive and culturally significant application is in the food domain. For over a millennium, dating back to the Tang Dynasty, an aqueous extract of VBTL has been traditionally used as a natural pigment to transform rice into “black rice” (*Wu mi*) [16], a ceremonial dish consumed during festivals in regions such as Jiangsu, Zhejiang, and the She ethnic community (Figure A1). This enduring culinary practice provides empirical evidence for the historical acceptance and safety of VBTL as a food ingredient. Furthermore, VBTL has already established certain applications in the field of functional foods, including commercial leaf powder for *Wu mi*, VBTL-derived tea products, instant tea bags, and dietary supplements [17]. These practical applications further support its safety profile. Modern scientific analyses have revealed that VBTL is abundant in bioactive constituents, primarily polysaccharides, flavonoids, and triterpenoids [17], which are associated with various health benefits such as antioxidant, hypoglycemic, anti-inflammatory, and antimicrobial effects [18,19,20]. These properties underpin its current applications in food preservation, natural pigment extraction, and traditional medicine for treating inflammation, diarrhea, and skin lesions [21].

Although the in vitro antioxidant activity of VBTL has been reported, its potential anti-aging efficacy, which is directly relevant to the development of functional food ingredients for promoting healthy aging, remains largely unexplored. Notably, the underlying molecular mechanisms and the key bioactive compounds responsible for these effects have yet to be elucidated. Therefore, this study aimed to systematically evaluate the anti-aging effects of VBTL extracts, a material derived from a historically edible plant, using multiple models. To this end, we employed models including a D-gal-induced aging mouse model, a *Caenorhabditis elegans* model, and an H_2_O_2_-induced cellular senescence model. We further sought to identify its active constituents and elucidate the mechanism through the Keap1-Nrf2 pathway. This research provides scientific evidence to support the development of VBTL as a promising, safe, and effective natural source for anti-aging functional foods and nutraceuticals.

## 2. Materials and Methods

### 2.1. Chemicals and Reagents

D-Galactose was purchased from Coolaber Technology Co., Ltd. (Beijing, China). Antioxidant enzyme kits for SOD, CAT, and GSH-Px, along with the MDA assay kit, were obtained from Nanjing Jiancheng Bioengineering Institute (Nanjing, China). The bicinchoninic acid (BCA) protein assay kit was acquired from Beyotime Biotechnology Co., Ltd. (Shanghai, China). The ChamQ Universal SYBR qPCR Master Mix and HiScript IV All-in-One Ultra RT SuperMix were provided by Nanjing Vazyme Biotech Co., Ltd. (Nanjing, China). 5-fluorouracil (5-FUDR) was supplied by Shanghai Acmec Biochemical Technology Co., Ltd. (Shanghai, China). Xanthotoxol was purchased from TargetMol Technology Co., Ltd. (Wellesley Hills, MA, USA). The Cell Counting Kit-8 (CCK-8) was obtained from GLPBIO Technology Co., Ltd. (Montclair, CA, USA). 2′,7′-Dichlorodihydrofluorescein diacetate (DCFH-DA, 98%) was procured from Sigma-Aldrich Trading Co., Ltd. (Shanghai, China). Cellular Senescence β-Galactosidase Staining Kit was acquired from Beyotime Biotechnology Co., Ltd. (Shanghai, China). The primary antibody against Nrf2 was sourced from Cell Signaling Technology, Inc. Antibodies against NQO1 and HO-1 were provided by Wuhan Sanying Biotechnology Co., Ltd. (Wuhan, China).

### 2.2. Preparation of VBTL Extracts and Determination of In Vitro Antioxidant Capacity

Dried *Vaccinium bracteatum* Thunb. leaves (VBTL) were ground into a fine powder. The extraction was performed by refluxing the powder with distilled water at a solid-to-liquid ratio of 1:30 (*w*/*v*) at 100 °C for 3 h. The mixture was filtered, and the residue was re-extracted once under identical conditions. Combined filtrates were centrifuged, and the supernatant was collected and concentrated under reduced pressure. The concentrated aqueous extract was then lyophilized to yield the final VBTL extracts in powder form.

The in vitro antioxidant capacity of the VBTL extracts was evaluated by measuring their 2,2′-azino-bis(3-ethylbenzothiazoline-6-sulfonic acid) (ABTS) radical inhibition rate, 2,2-diphenyl-1-picrylhydrazyl (DPPH) radical scavenging activity, and reducing power, with vitamin C (Vc) used as a positive control. ABTS inhibition [22], DPPH scavenging [23], and reducing power [24] were conducted following previously reported methods with minor modifications.

### 2.3. Animal Experimental Design

Seventy-two male Balb/c mice (20 ± 2 g) were obtained from Beijing Vital River Laboratory Animal Technology Co., Ltd. (Beijing, China). All mice were housed in the Animal Center of Jinan University under controlled conditions: temperature 23 ± 2 °C, humidity 55 ± 5%, and a 12-h light/dark cycle. After one week of acclimatization, animals were randomly allocated to six groups (*n* = 12 per group): control group (Con), model group (Mod), Vc-treated group (Vc), and three VBTL-treated groups at low, medium, and high doses (VBTL-L, VBTL-M, VBTL-H). The sample size was determined based on previous studies using similar experimental models [25,26]. Except for the control group, which was administered an equal volume of physiological saline (0.9%), the remaining groups were injected intraperitoneally with D-galactose (200 mg/kg body weight) every morning. In the afternoon, the model group was given 0.9% saline by oral gavage, whereas the VBTL-treated groups received VBTL solutions at 100, 200, and 400 mg/kg body weight, respectively, via the same route. The doses were determined based on previous studies [27,28]. The animal experimental protocol was reviewed and approved by the Experimental Animal Ethics Committee of Jinan University (Guangzhou, China; Approval No. IACUC-20240923-07) and was conducted in strict accordance with the guidelines of the university’s Institutional Animal Care and Use Committee. Body weights were recorded weekly. After 8 consecutive weeks of intervention, the mice were fasted (with water withheld) for 24 h following the final administration, then anesthetized and euthanized. Blood and tissue specimens were collected for subsequent analysis.

### 2.4. Determination of Biochemical Indicators

Tissues (0.1 g) were mechanically homogenized in physiological saline to obtain a 10% (*w*/*v*) homogenate. Following centrifugation at 3500 rpm for 10 min at 4 °C, the resulting supernatant was collected for subsequent biochemical assays. Both tissue supernatants and serum samples were analyzed for antioxidant-related indicators using commercial assay kits according to the manufacturer’s instructions. The protein concentration of the samples was determined using a BCA protein quantification kit.

### 2.5. Histopathological Analysis

Liver tissues collected from the mice were immediately fixed in 4% paraformaldehyde. Subsequently, the tissues were processed for paraffin embedding, sectioned, and stained with hematoxylin and eosin (H&E) following standard protocols. The histopathological morphology of the tissues from each group was subsequently observed under a light microscope.

### 2.6. Effect on Nrf2 Pathway Gene Expression in Mouse Liver

To determine mRNA expression in the liver, total RNA was extracted using the Trizol reagent. Subsequently, the RNA was reverse-transcribed into complementary DNA (cDNA) using the HiScript IV All-in-One Ultra RT SuperMix for qPCR kit purchased from Nanjing Vazyme Biotech Co., Ltd. (Nanjing, China). Gene-specific primers were custom-synthesized by Sangon Biotech Co., Ltd. (Shanghai, China). Quantitative real-time polymerase chain reaction (qRT-PCR) was performed to quantify mRNA expression levels using the primer sets listed in Table 1. The thermal cycling profile consisted of an initial denaturation at 95 °C for 30 s, followed by 40 amplification cycles (95 °C for 10 s, 60 °C for 30 s). A melt curve analysis was then incorporated under the following conditions: 95 °C for 15 s, 60 °C for 1 min, and a final ramp to 95 °C for 15 s. Relative mRNA expression levels were quantified using the comparative 2^−ΔΔCt^ method, with *GAPDH* serving as the internal reference gene for normalization.

### 2.7. Cultivation and Synchronization of Caenorhabditis elegans

The wild-type *Caenorhabditis elegans* N2 were maintained on nematode growth medium (NGM) plates seeded with *Escherichia coli* OP50 at 20 °C and 60% relative humidity. When the majority of the nematodes reached the L4 larval stage, synchronization was performed under aseptic conditions. Nematodes were collected in centrifuge tubes containing 2 mL of M9 buffer (prepared by dissolving 0.3 g KH_2_PO_4_, 0.6 g NaCl, 1.512 g Na_2_HPO_4_·12H_2_O, and 0.1 mL of 1 M MgSO_4_ in distilled water to a final volume of 100 mL). After centrifugation at 3000 rpm for 2 min and settling for 1 min, the supernatant was carefully removed and discarded. Subsequently, each tube received 1 mL of freshly prepared cracking solution (composed of 5 mL NaClO, 1.25 mL 10 M NaOH, and 18.5 mL distilled water) and was incubated for 5 min to lyse the adult nematodes. After another centrifugation at 3000 rpm for 1 min, the supernatant was carefully removed, and the pelleted eggs were retained. The resultant pellet was subjected to triple washing with 1.5 mL aliquots of M9 buffer, with intervening centrifugation at 3000 rpm for 1 min following each wash cycle. Upon completion of the aforementioned processing steps, the nematode eggs were subsequently transferred onto prepared NGM plates previously seeded with *E. coli* OP50 as a food source. The plates were incubated under constant conditions (20 °C, 60% humidity) for 56 h to obtain synchronized L4-stage *C. elegans*. Based on preliminary experiments, two concentrations of VBTL extracts that did not affect normal nematode growth were selected for subsequent experiments: 1 mg/mL as the low-dose group (VBTL-L) and 5 mg/mL as the high-dose group (VBTL-H).

### 2.8. Antioxidant Enzyme Activities and MDA Levels in C. elegans

Experimental groups were designated as follows: Control (Con), Vitamin C (Vc, positive control, 1 mg/mL), VBTL low-dose (VBTL-L, 1 mg/mL), and VBTL high-dose (VBTL-H, 5 mg/mL). For each group, a 500 μL aliquot of *E. coli* OP50 suspension was evenly spread onto 90 mm NGM plates and allowed to dry. Subsequently, 500 μL of the corresponding sterile sample solution was evenly applied to the bacterial lawn. Synchronized L4-stage *C. elegans* were transferred onto these plates, with three replicate plates per group and approximately 500 nematodes per plate. After 3–5 days of incubation under controlled conditions (20 °C, 60% relative humidity), nematodes from each treatment group were collected, washed with M9 buffer, and centrifuged at 1000× *g* for 1 min. The resulting nematode pellet (≈500 μL) was homogenized in 800 μL of ice-cold physiological saline using a tissue homogenizer at 60 Hz for 1 min. The homogenate was then centrifuged (4000 rpm, 2 min, 4 °C), and the resulting supernatant was collected. Protein content was quantified using a BCA assay kit. The activities of SOD and CAT, as well as MDA levels, were measured using their respective commercial assay kits. Final results were normalized to total protein content and expressed as U/mg prot for SOD and CAT, and as nmol/mg prot for MDA.

### 2.9. Measurement of Lipofuscin Levels

The experimental groups were consistent with those described in Section 2.8. Briefly, 100 μL of each sample solution at the designated concentration was applied to 60 mm NGM plates supplemented with 150 μM 5-FUDR and pre-inoculated with *E. coli* OP50. Negative control conditions were established using sterile distilled water. Synchronized L4-stage *C. elegans* were randomly allocated into four groups, each with triplicate plates of 30 nematodes. The nematodes were transferred onto the prepared plates and cultured for 10 days. Subsequently, nematodes were collected into 2 mL tubes pre-filled with M9 buffer and immediately subjected to a 60 °C water bath for 1 min. The nematodes were then immobilized on microscope slides coated with 2% agarose. Fluorescence micrographs were captured using an inverted epifluorescence microscopy system. Subsequent quantitative analysis of fluorescence intensity was performed using ImageJ software (version 1.54 g).

### 2.10. Determination of the Effect of VBTL Extracts on Lifespan and Reproductive Capacity

#### 2.10.1. Lifespan Assay

The experimental groups were the same as described in Section 2.8. For each group, 100 µL of an *E. coli* OP50 suspension was spread onto NGM agar plates containing 5-FUDR. Subsequently, 100 µL of each sample solution at the corresponding concentration (sterile distilled water for the control group) was applied to the plates. Synchronized L4-stage *C. elegans* were randomly selected and transferred onto the prepared plates, with three replicate plates per group and 30 nematodes per plate. The first day of transfer was recorded as day 0. Nematode survival was monitored daily until the last individual in each group had died.

#### 2.10.2. Fertility Experiment

The experimental groups consisted of Control (Con), Vitamin C (Vc, positive control), VBTL low-dose (VBTL-L), and VBTL high-dose (VBTL-H). For each experimental group, *E. coli* OP50 suspension (100 μL) and the corresponding sterile sample solution were consecutively applied onto 60 mm NGM agar plates. Three plates were prepared per group, and a single synchronized L4-stage nematode was placed onto each plate. The transfer day was designated as day 0. Every 24 h, the adult nematode was transferred to a fresh plate of the same treatment. The eggs laid on the previous plate were counted 3 days after the initial transfer (i.e., counting eggs laid on day 1 on day 3). This procedure of daily transfer and subsequent egg counting was repeated until the nematode ceased egg-laying. Three biologically independent replicates were included per group.

### 2.11. Screening of Key Metabolites and Molecular Docking Analysis

To identify the potential anti-aging active compounds in the VBTL extracts, an untargeted metabolomics analysis was performed on mouse serum samples. After pre-treatment, serum samples were analyzed using ultra-performance liquid chromatography coupled with tandem mass spectrometry (UPLC-MS/MS). Raw mass spectrometric data were converted to mzXML format using the ProteoWizard software (version 3.0). Peak extraction, peak alignment, and retention-time correction were performed with the XCMS package. Peaks with a missing rate > 50% were removed, and any remaining missing values were imputed using the k-nearest neighbors (KNN) method. Peak areas were subsequently normalized by the support vector regression (SVR) algorithm. Metabolite identification was based on HMDB, PubChem, KEGG and METLIN databases and the metDNA method. Processed data were analyzed by means of principal component analysis (PCA) and orthogonal partial least squares-discriminant analysis (OPLS-DA). Differentially abundant metabolites were identified based on the following criteria: variable importance in projection (VIP) > 1 and *p*-value < 0.05, followed by Spearman correlation analysis to assess their interrelationships.

To evaluate the binding affinity of the predicted active compounds to the target protein, molecular docking was performed. The three-dimensional structure files of the candidate compounds were obtained from the PubChem database. The crystal structure of the Kelch-like ECH-associated protein 1 (Keap1) protein (PDB ID: 6YTM) was retrieved from the RCSB Protein Data Bank. Molecular docking was executed using AutoDockTools 1.5.7, and the binding energies were predicted. Finally, the interaction patterns between the compounds and Keap1 were visualized using PyMOL software (version 3.1.0).

### 2.12. Cell Culture and Cytotoxicity Assay

HepG2 cells were cultured in Dulbecco’s Modified Eagle Medium (DMEM) supplemented with 10% (*v*/*v*) fetal bovine serum (FBS) and 1% (*v*/*v*) penicillin-streptomycin. Incubation conditions comprised 37 °C and 5% CO_2_ in a humidified environment. Cell viability was assessed through CCK-8 assay [29]. To determine the cytotoxicity of xanthotoxol, cells were seeded into 96-well plates at 1 × 10^4^ cells/well and permitted to adhere for 24 h. The culture medium was subsequently aspirated and replenished with fresh complete medium spiked with escalating concentrations of xanthotoxol (0–32 μmol/L). Following an additional 24 h of treatment, CCK-8 reagent (10 μL) was added to each well. After 1 h of incubation at 37 °C, the absorbance at 450 nm was measured using a microplate spectrophotometer. To determine the appropriate concentration of H_2_O_2_ for inducing oxidative stress, HepG2 cells were similarly seeded. After 24 h, the medium was replaced with freshly prepared formulations containing graded concentrations of H_2_O_2_ (0–800 μmol/L). Following 5 h of exposure, cell viability was measured using the CCK-8 assay.

### 2.13. Measurement of Intracellular ROS Levels and Senescence-Associated β-Galactosidase Staining

ROS levels were quantified using the fluorescent probe DCFH-DA. HepG2 cells were seeded in 96-well plates (1 × 10^4^ cells/well). After 24 h of incubation, cells were pretreated with different concentrations of xanthotoxol (2, 4, and 8 μmol/L) for 24 h and then exposed to 500 μmol/L H_2_O_2_ for 5 h. The model group was exposed to 500 μmol/L H_2_O_2_ alone for 5 h to induce premature senescence, while the control group received culture medium only. After treatment, cells were washed twice with phosphate-buffered saline (PBS) and incubated with serum-free DMEM containing 10 μmol/L DCFH-DA probe at 37 °C in the dark for 20–30 min. The probe solution was subsequently removed, and cells were washed three times with PBS. Fluorescence signal intensity was subsequently quantified via microplate spectrophotometry at 488 nm excitation and 535 nm emission wavelengths (λex = 488, λem = 535).

Senescent cells were specifically identified by detecting senescence-associated β-galactosidase (SA-β-gal) activity [30]. HepG2 cells were plated into 6-well plates (2 × 10^5^ cells/well) and incubated for 24 h. Subsequently, cells were pretreated with xanthotoxol (2, 4, and 8 μmol/L) for 24 h, followed by exposure to 500 μmol/L H_2_O_2_ for 5 h. The model group was treated with 500 μmol/L H_2_O_2_ for 5 h, while the control group received culture medium only. Subsequently, SA-β-gal activity was detected using a cellular senescence β-galactosidase staining kit in accordance with the manufacturer’s instructions.

### 2.14. Determination of Cellular Antioxidant Indicators

Cells were seeded in 6-well plates and subjected to experimental procedures outlined in Section 2.13. After removing the culture medium, HepG2 cells were collected and subjected to lysis on ice using RIPA lysis buffer. Intracellular protein content was quantified using a BCA assay kit. Subsequently, intracellular SOD activity and MDA content were detected.

### 2.15. RNA Extraction and RT-qPCR

The methodology was the same as in Section 2.6. Primer sets for each gene are listed in Table 2.

### 2.16. Western Blot Analysis

HepG2 cells were collected and lysed on ice using RIPA lysis buffer. Protein concentrations were determined with a BCA assay kit. Equal amounts of protein were separated by means of 10% sodium dodecyl sulfate–polyacrylamide gel electrophoresis (SDS-PAGE) and subsequently transferred onto polyvinylidene fluoride (PVDF) membranes. The membranes were blocked with 5% skimmed milk at room temperature for 1 h and then incubated with specific primary antibodies at 4 °C overnight. After washing with Tris-buffered saline containing 0.1% Tween 20 (TBST), membranes were incubated for 2 h with appropriate horseradish peroxidase (HRP)-conjugated secondary antibodies (diluted 1:5000). After a final wash with TBST, protein signals were detected using an enhanced chemiluminescence (ECL) substrate. Band signal intensities were quantified using ImageJ software.

### 2.17. Statistical Analysis

All data are expressed as the mean ± standard deviation (SD). GraphPad Prism software (version 9.5) was used for data processing and graphical visualization. Statistical analysis was performed using one-way ANOVA followed by Dunnett’s post hoc test to correct for multiple comparisons. Statistical significance was defined as *p* < 0.05. Each experiment comprised at least three independent biological replicates.

## 3. Results

### 3.1. In Vitro Antioxidant Activity

**Figure 1:**
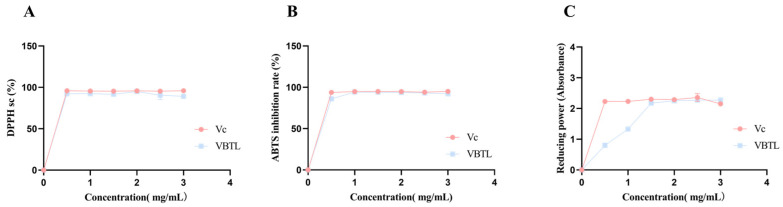
Determination of the DPPH-scavenging activity, ABTS-inhibitory capacity and Reducing power of VBTL extracts. (**A**) The DPPH-scavenging activity of VBTL extracts. (**B**) The ABTS-inhibitory capacity of VBTL extracts. (**C**) The reducing power of VBTL extracts.

The in vitro antioxidant capacity of VBTL extracts was evaluated by means of DPPH, ABTS, and reducing power assays. As shown in Figure 1, the VBTL extracts exhibited potent antioxidant activity in vitro. DPPH radical scavenging activity reached 89.14 ± 2.20%, while ABTS radical inhibition was 92.15 ± 0.38%. The reducing power of the extracts was 2.28 ± 0.07. In comparison, the positive control vitamin C (Vc) displayed values of 95.94 ± 0.51% for DPPH scavenging, 95.06 ± 0.15% for ABTS inhibition, and 2.149 ± 0.033 for reducing power. These results indicated that the VBTL extracts possessed significant free radical scavenging capacity and reducing ability, comparable to the reference antioxidant Vc.

### 3.2. VBTL Extracts Attenuated Oxidative Stress in D-Galactose-Induced Aging Mice

**Figure 2:**
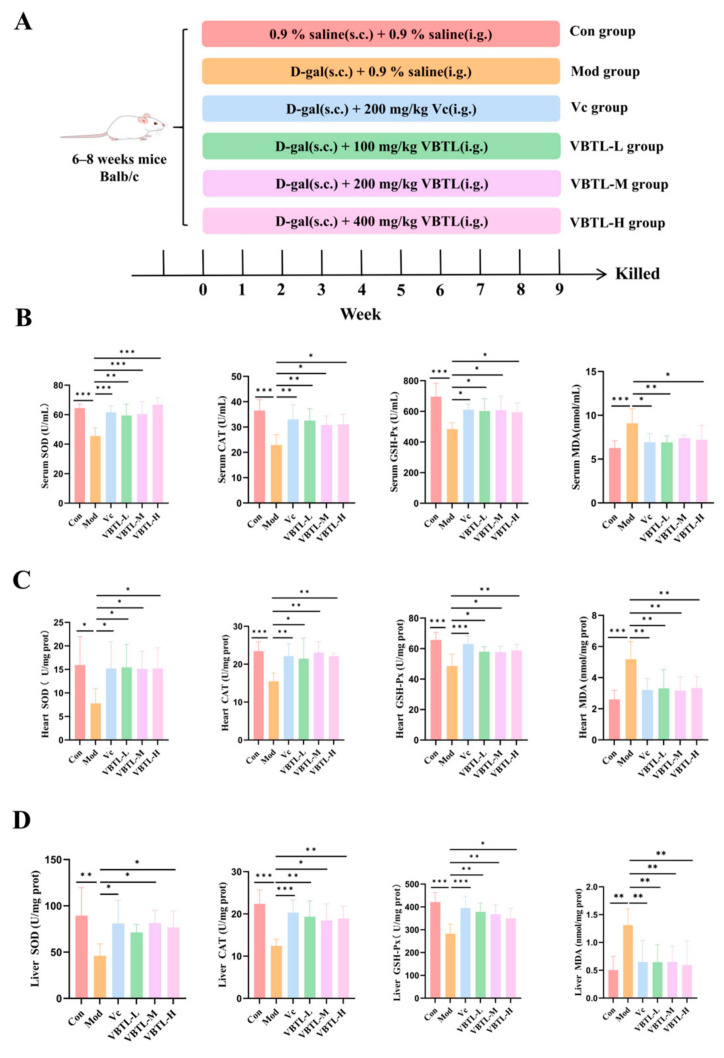
VBTL extracts mitigated oxidative stress injury in serum, heart and liver of aging mice induced by D-galactose. (**A**) Animal experiment flow chart. (**B**) Serum. (**C**) Heart. (**D**) Liver. Control group (Con); D-gal-induced aging model group (Mod); D-gal-induced aging mice treated with vitamin C at a dosage of 200 mg/kg (Vc); D-gal-induced aging mice treated with VBTL at a dosage of 100 mg/kg (VBTL-L); D-gal-induced aging mice treated with VBTL at a dosage of 200 mg/kg (VBTL-M); D-gal-induced aging mice treated with VBTL at a dosage of 400 mg/kg (VBTL-H). Statistical significance: * *p* < 0.05, ** *p* < 0.01, *** *p* < 0.001.

To evaluate the in vivo anti-aging efficacy, we employed a D-gal-induced aging mouse model. The activities of key antioxidant enzymes and the level of MDA were measured in the serum, heart, and liver tissues of the mice. Compared with the control group, the model group exhibited significantly reduced activities of SOD, CAT, and GSH-Px, along with elevated MDA level (Figure 2B–D), indicating successful induction of oxidative stress by D-gal. In contrast, both the Vc-treated group and the VBTL-treated groups exhibited significantly elevated activities of SOD, CAT, and GSH-Px, as well as a significantly reduced MDA level, compared with the model group. These results demonstrated that VBTL extracts effectively restored the redox balance and alleviated oxidative stress in D-gal-induced aging mice.

### 3.3. Histopathological Analysis of Liver Tissues

**Figure 3:**
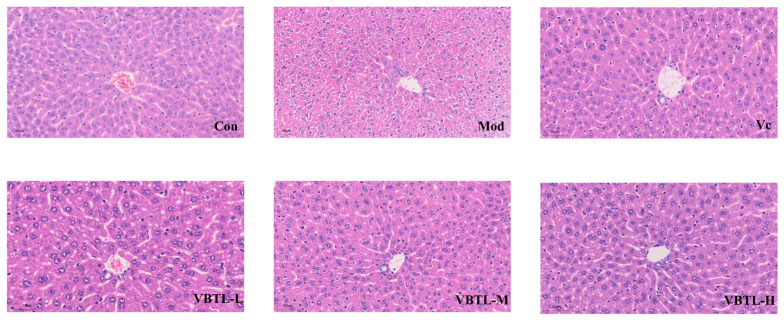
Effect of VBTL extracts on D-gal-induced mouse liver histology, revealed by haematoxylin and eosin (H&E) staining (400×). Control group (Con); D-gal-induced aging model group (Mod); D-gal-induced aging mice treated with vitamin C at a dosage of 200 mg/kg (Vc); D-gal-induced aging mice treated with VBTL at a dosage of 100 mg/kg (VBTL-L); D-gal-induced aging mice treated with VBTL at a dosage of 200 mg/kg (VBTL-M); D-gal-induced aging mice treated with VBTL at a dosage of 400 mg/kg (VBTL-H).

To evaluate the protective effect of VBTL at the tissue level, we performed histopathological analysis of liver sections. As shown in Figure 3, the liver tissues from the control group exhibited a normal architecture with intact hepatocytes, uniform cytoplasm, and clear nuclei and nucleoli. In contrast, the D-gal-induced model group exhibited significant pathological damage, characterized by hydropic degeneration of hepatocytes (cell swelling with pale and loosely arranged cytoplasm), obvious inflammatory cell infiltration around the central vein, disarranged hepatic cords, and severe disruption of the normal liver structure. However, treatment with either Vc or various doses of VBTL extracts markedly attenuated these pathological alterations, leading to a significant improvement in liver histoarchitecture.

### 3.4. VBTL Extracts Upregulated Liver Expression of Genes in the Nrf2 Pathway

**Figure 4:**
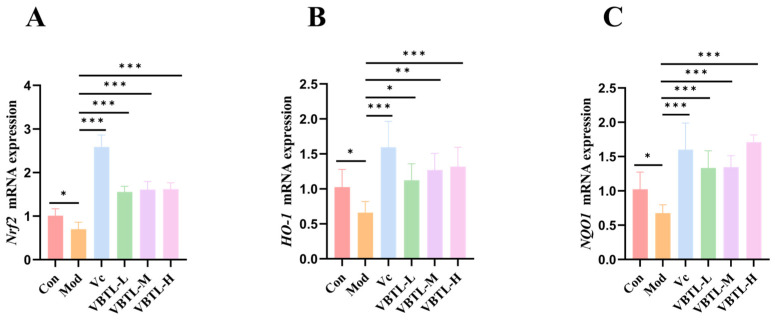
Effect of VBTL extracts on *Nrf2*, *HO-1*, and *NQO1* gene expression. (**A**) *Nrf2*. (**B**) *HO-1*. (**C**) *NQO1*. Control group (Con); D-gal-induced aging model group (Mod); D-gal-induced aging mice treated with vitamin C at a dosage of 200 mg/kg (Vc); D-gal-induced aging mice treated with VBTL at a dosage of 100 mg/kg (VBTL-L); D-gal-induced aging mice treated with VBTL at a dosage of 200 mg/kg (VBTL-M); D-gal-induced aging mice treated with VBTL at a dosage of 400 mg/kg (VBTL-H). Statistical significance: * *p* < 0.05, ** *p* < 0.01, *** *p* < 0.001.

Nrf2 is a crucial transcription factor that regulates the expression of genes involved in antioxidant defense, anti-inflammatory responses, and detoxification [31]. The mRNA expression levels of *Nrf2*, *NQO1*, and *HO-1* in liver tissues were significantly downregulated in the model group compared with the control group (*p* < 0.05). In contrast, treatment with VBTL extracts significantly upregulated the mRNA expression levels of these genes relative to the model group (Figure 4A–C) (*p* < 0.05). These findings indicated that VBTL extracts activated the Nrf2 signaling pathway, thereby enhancing the transcriptional level of downstream antioxidant genes.

### 3.5. Effect of VBTL Extracts on Antioxidant Enzyme Activities and MDA Levels in C. elegans

**Figure 5:**
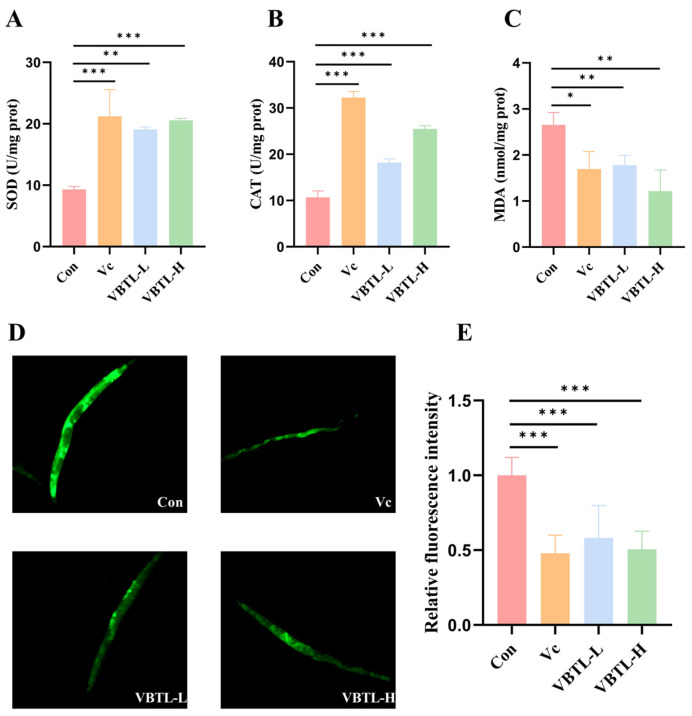
Effect of VBTL extracts on antioxidant enzymes, MDA content and lipofuscin accumulation in *C. elegans*: (**A**) SOD. (**B**) CAT. (**C**) MDA. (**D**) Detection of lipofuscin autofluorescence in *C. elegans.* (**E**) Relative fluorescence intensity. Con (Control group); Vc (treatment with 1 mg/mL vitamin C); VBTL-L (treatment with 1 mg/mL VBTL); VBTL-H (treatment with 5 mg/mL VBTL). Statistical significance: * *p* < 0.05, ** *p* < 0.01, *** *p* < 0.001.

In the *C. elegans* model, treatment with VBTL extracts significantly enhanced the organism’s antioxidant defense capacity. Compared with the control group, treatment with both low- and high-dose VBTL extracts markedly enhanced SOD activity by 104.69% and 120.56%, respectively, and elevated CAT activity by 70.11% and 138.39% (*p* < 0.05) (Figure 5A,B). Furthermore, the MDA content in nematodes from the VBTL-L and VBTL-H groups was significantly reduced by 32.97% and 54.25%, respectively, compared with the control group (*p* < 0.05). Notably, the MDA level in the VBTL-H group was significantly decreased compared with the Vc group (Figure 5C). These results demonstrated that VBTL extracts significantly enhanced antioxidant enzyme activities and alleviated lipid peroxidation damage in *C. elegans*.

### 3.6. Effect of VBTL Extracts on Lipofuscin Levels in C. elegans

Lipofuscin, a well-established biomarker of aging, exhibits progressive accumulation with age [32]. Compared with the control group, both Vc and VBTL treatments significantly reduced the autofluorescence intensity of lipofuscin in the nematodes (*p* < 0.05) (Figure 5D,E). The reduction was 52.01% in the Vc group, 41.82% in the VBTL-L group, and 49.43% in the VBTL-H group. These results demonstrated that VBTL extracts significantly reduced lipofuscin accumulation in *C. elegans*, thereby delaying aging in the nematodes.

### 3.7. Effect of VBTL Extracts on Lifespan and Reproductive Toxicity in C. elegans

**Figure 6:**
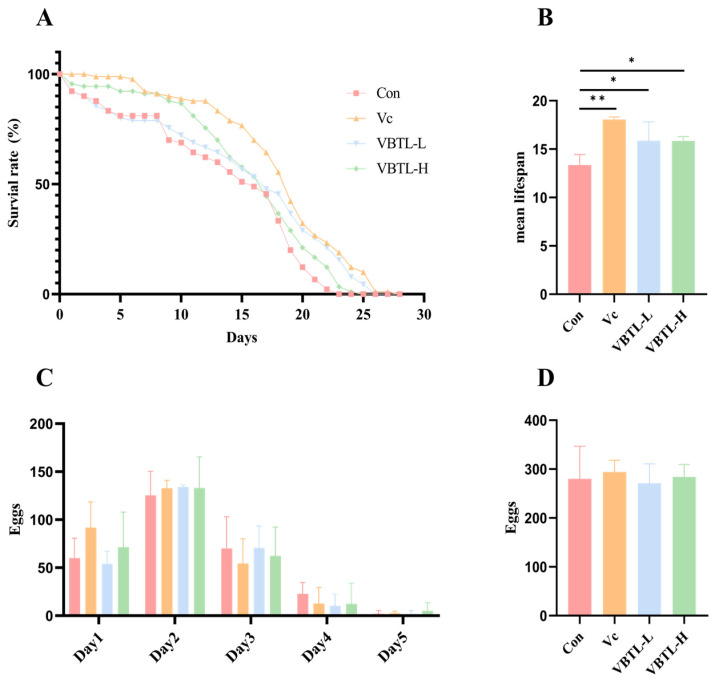
Effect of VBTL extracts on the lifespan and oviposition ability of *C. elegans.* (**A**) The lifespan curve of *C. elegans.* (**B**) The mean lifespan of *C. elegans.* (**C**) Daily egg production of *C. elegans.* (**D**) Total egg production of *C. elegans.* Con (Control group); Vc (treatment with 1 mg/mL vitamin C); VBTL-L (treatment with 1 mg/mL VBTL); VBTL-H (treatment with 5 mg/mL VBTL). Statistical significance: * *p* < 0.05, ** *p* < 0.01.

Lifespan is a key quantitative indicator of aging [33]. As shown in Figure 6A,B, the mean lifespan of nematodes in the control group was 13.36 ± 1.08 days. In contrast, treatment with low- and high-concentration VBTL extracts significantly extended the mean lifespan to 15.87 ± 1.95 days and 15.84 ± 0.4 days, respectively, representing extensions of 18.82% and 18.57% compared with the control group. There was no significant difference in lifespan between the two VBTL-treated groups (*p* > 0.05). These results demonstrated that VBTL extracts significantly extended the lifespan of *C. elegans*.

A decline in fecundity is often associated with lifespan extension [34]. Therefore, we assessed whether VBTL extracts exhibited reproductive toxicity. As shown in Figure 6C,D, the mean brood size in the control group (without VBTL treatment) was 280.00 ± 64.90 eggs. After treatment with low- and high-concentration VBTL extracts, the brood sizes were 271.00 ± 39.00 and 284.00 ± 25.63 eggs, respectively, with no significant differences observed among the groups (*p* > 0.05). These results indicated that VBTL extracts extended lifespan without adversely affecting the reproductive capacity of *C. elegans*.

### 3.8. Screening of Key Metabolites and Determination of Cell Viability

**Figure 7:**
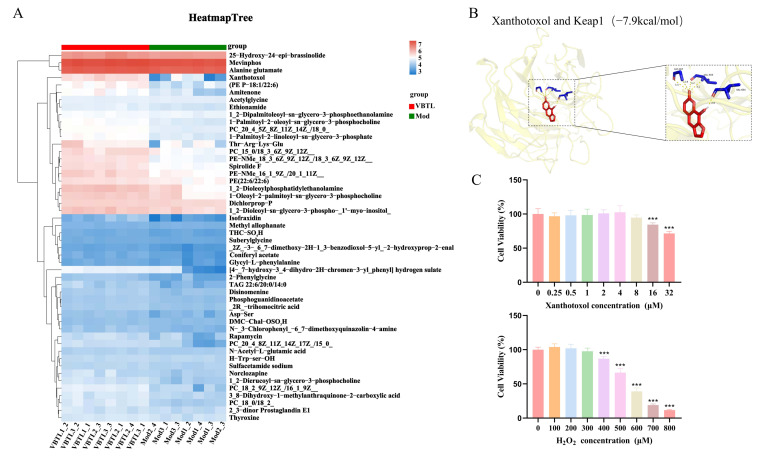
Screening of key metabolites and the effect of xanthotoxol and H_2_O_2_ on the viability of HepG2 cells. (**A**) Heatmap Tree. (**B**) Molecular docking of xanthotoxol and Keap1. (**C**) Effects of xanthotoxol and H_2_O_2_ on HepG2 cell viability. In subfigure B, the red substance represents xanthotoxol, the blue represents the relevant amino acid residues, and the yellow dashed lines indicate hydrogen bonds. Results are presented as means ± standard deviation (SD, *n* = 6). *** Significant differences from control group at *p* < 0.001.

To identify the potential anti-aging bioactive constituents, an untargeted metabolomics analysis of mouse serum was conducted. This analysis revealed that 255 metabolites were significantly upregulated in the VBTL-treated group compared with the model group. Spearman correlation analysis further identified 50 metabolites that were highly correlated with the oxidation indicators (Figure 7A). Among these 50 correlated metabolites, the linear furanocoumarin xanthotoxol (Xan) was present at a higher level in the VBTL-treated group than in the model group (*p* < 0.05). Furthermore, it was also identified as the most abundant compound among these correlated metabolites within the VBTL extracts. Given its reported antioxidant and anti-inflammatory activities [35,36], xanthotoxol was prioritized as the lead candidate for mechanistic investigation.

Keap1 is a key regulator of the Nrf2 signaling pathway, promoting the ubiquitination and degradation of Nrf2 through direct binding. Molecular docking was performed to evaluate the potential binding between xanthotoxol and the Keap1 protein. The results indicated favorable binding affinity between xanthotoxol and Keap1. Xanthotoxol was predicted to bind to Keap1 at the residues GLY-367, VAL-606, and VAL-604 (Figure 7B). A binding affinity of ≤−5.0 kcal/mol is generally considered to indicate a stable binding conformation. Our docking model revealed a binding affinity of −7.9 kcal/mol for the xanthotoxol-Keap1 complex. These findings suggested that xanthotoxol, a constituent of VBTL, may directly bind to Keap1, potentially blocking its degradation effect on Nrf2 and thereby activating the Nrf2-mediated protective pathway.

To investigate the role of xanthotoxol in alleviating oxidative stress-induced senescence through the Nrf2 pathway, a series of in vitro experiments were conducted. The CCK-8 assay results confirmed that xanthotoxol had no significant effect on cell viability at concentrations up to 8 μmol/L (Figure 7C). Therefore, concentrations of 2, 4, and 8 μmol/L xanthotoxol were selected for subsequent experiments. Meanwhile, H_2_O_2_ exposure induced concentration-dependent reductions in cell viability. Significant cytotoxicity was observed upon exceeding 300 μmol/L H_2_O_2_ (*p* < 0.05). After 5 h of treatment with 500 μmol/L H_2_O_2_, cell viability was reduced to 66.45%, consistent with previously established senescence-inducing conditions [37]. Consequently, 500 μmol/L H_2_O_2_ was used to establish the cellular senescence model for further studies.

### 3.9. Effect of Xanthotoxol on SA-β-Gal Activity and Intracellular ROS Levels

**Figure 8:**
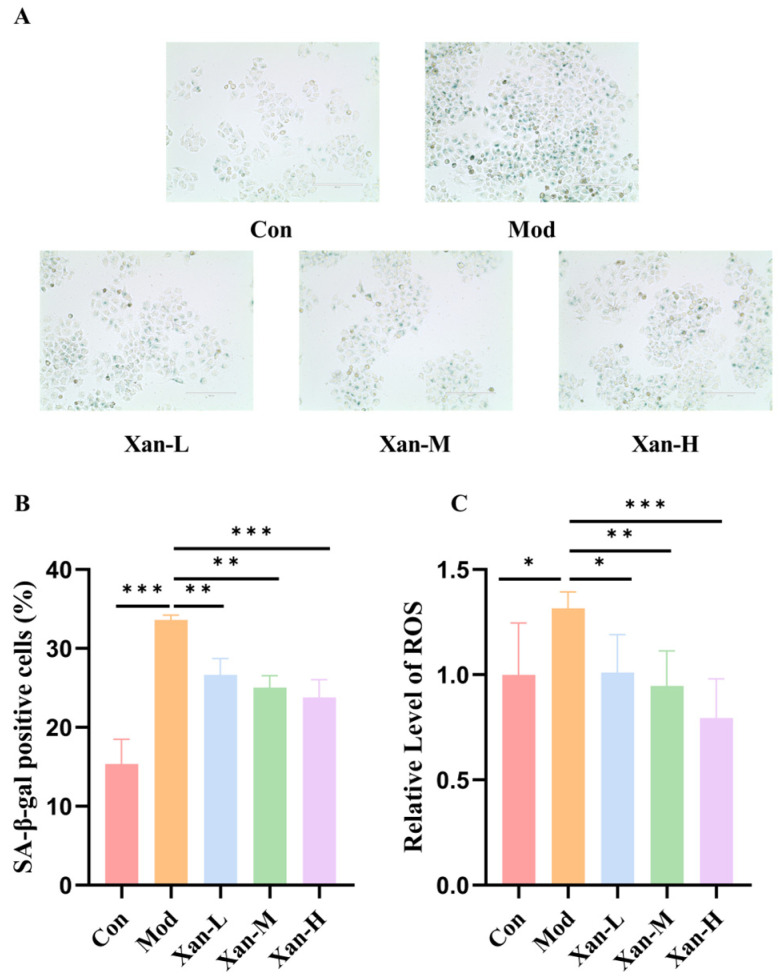
Effect of xanthotoxol on senescence-associated β-galactosidase activity and ROS levels. (**A**) SA-β-gal positive cells observed by means of light microscopy (20×). (**B**) The percentages of SA-β-gal positive cells are reported as means ± SD (*n* = 3). (**C**) Relative level of ROS. Control group (Con); H_2_O_2_-induced aging model group (Mod); H_2_O_2_-induced cells treated with 2 μmol/L xanthotoxol (Xan-L); H_2_O_2_-induced cells treated with 4 μmol/L xanthotoxol (Xan-M); H_2_O_2_-induced cells treated with 8 μmol/L xanthotoxol (Xan-H). Statistical significance: * *p* < 0.05, ** *p* < 0.01, *** *p* < 0.001.

To validate whether the identified key constituent, xanthotoxol, directly confers anti-senescence effects, we examined its impact on SA-β-gal activity and ROS levels in H_2_O_2_-injured cells. Senescent cells were identified through SA-β-gal staining, with positive cells exhibiting characteristic blue–green coloration. Compared with the control group, the model group exhibited a 119.02% elevation in SA-β-gal positivity (*p* < 0.05), confirming successful induction of cellular senescence by H_2_O_2_ (Figure 8A,B). Treatment with different concentrations of xanthotoxol significantly reduced β-galactosidase activity compared with the model group. With increasing xanthotoxol concentration, the SA-β-gal positivity rates in Xan-L, Xan-M, and Xan-H groups were reduced by 20.76%, 25.51%, and 29.33%, respectively, demonstrating a dose-dependent alleviation of the senescent phenotype.

Regarding oxidative stress, H_2_O_2_ stimulation markedly enhanced intracellular ROS fluorescence intensity, while xanthotoxol treatment effectively suppressed ROS accumulation. Relative to the model group, intracellular ROS levels were significantly reduced by 23.21%, 27.99%, and 39.60% in Xan-L, Xan-M, and Xan-H groups (Figure 8C). These results demonstrated that xanthotoxol significantly alleviated H_2_O_2_-induced cellular senescence and inhibited intracellular ROS accumulation, with the highest concentration exerting the most pronounced effect. Therefore, the highest concentration of xanthotoxol (8 μmol/L) was selected for subsequent mechanistic investigations.

### 3.10. Effect of Xanthotoxol on Antioxidant Enzyme Activity, SASP Levels, and P53/P21/P16 mRNA Expression

**Figure 9:**
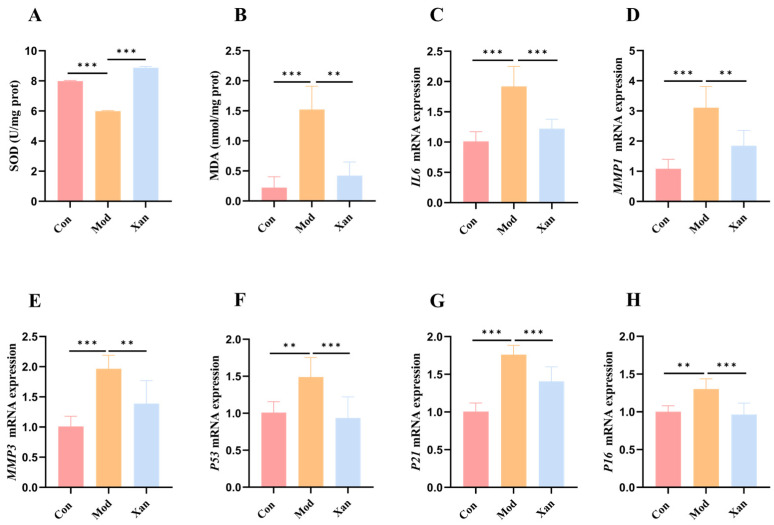
Effect of xanthotoxol on antioxidant enzymes, SASP levels and *P53*/*P21*/*P16* gene expression. (**A**) SOD. (**B**) MDA. (**C**) *IL6*. (**D**) *MMP1*. (**E**) *MMP3*. (**F**) *P53*. (**G**) *P21*. (**H**) *P16*. Control group (Con); H_2_O_2_-induced aging model group (Mod); H_2_O_2_-induced cells treated with 8 μmol/L xanthotoxol (Xan). Statistical significance: ** *p* < 0.01, *** *p* < 0.001.

To elucidate the mechanistic basis of its anti-aging action, we investigated the effects of xanthotoxol on oxidative defense, SASP, and core cell-cycle arrest pathways (*P53*/*P21*/*P16*). In the H_2_O_2_-induced HepG2 cellular senescence model, significant alterations in oxidative stress markers were observed. Relative to the control group, the model group exhibited a significant reduction in SOD activity and a significant elevation in MDA level, confirming that H_2_O_2_ induced oxidative stress damage. In contrast, the xanthotoxol-treated group demonstrated a significant enhancement in SOD activity and a significant reduction in MDA level compared with the model group (Figure 9A,B). These results indicated that xanthotoxol possessed antioxidative capacity and effectively mitigated the H_2_O_2_-induced redox imbalance.

H_2_O_2_ stimulation significantly upregulated the secretion levels of typical SASP factors, including interleukin-6 (IL-6), matrix metalloproteinase-1 (MMP1), and matrix metalloproteinase-3 (MMP3). Relative to the control group, the model group exhibited significant increases in the levels of these SASP factors by 89.80%, 186.29%, and 94.24%, respectively (*p* < 0.05). In contrast, xanthotoxol intervention significantly reduced the SASP levels compared with the model group (*p* < 0.05), with reductions of 36.34%, 40.49%, and 29.29%, respectively (Figure 9C–E).

Furthermore, oxidative stress induces cellular senescence through pathways that alter *P53*/*P21*/*P16* gene expression [38]. The mRNA expression levels of *P53*/*P21*/*P16* were significantly elevated in the model group relative to the control group (*p* < 0.05), by 47.49%, 75.00%, and 29.68%, respectively. Conversely, relative to the model group, xanthotoxol treatment significantly downregulated the expression of these senescence markers (*p* < 0.05), with reductions of 37.11%, 20.07%, and 25.86%, respectively (Figure 9F–H).

### 3.11. Xanthotoxol Exerted Anti-Aging Effects via Activation of the Nrf2 Pathway

**Figure 10:**
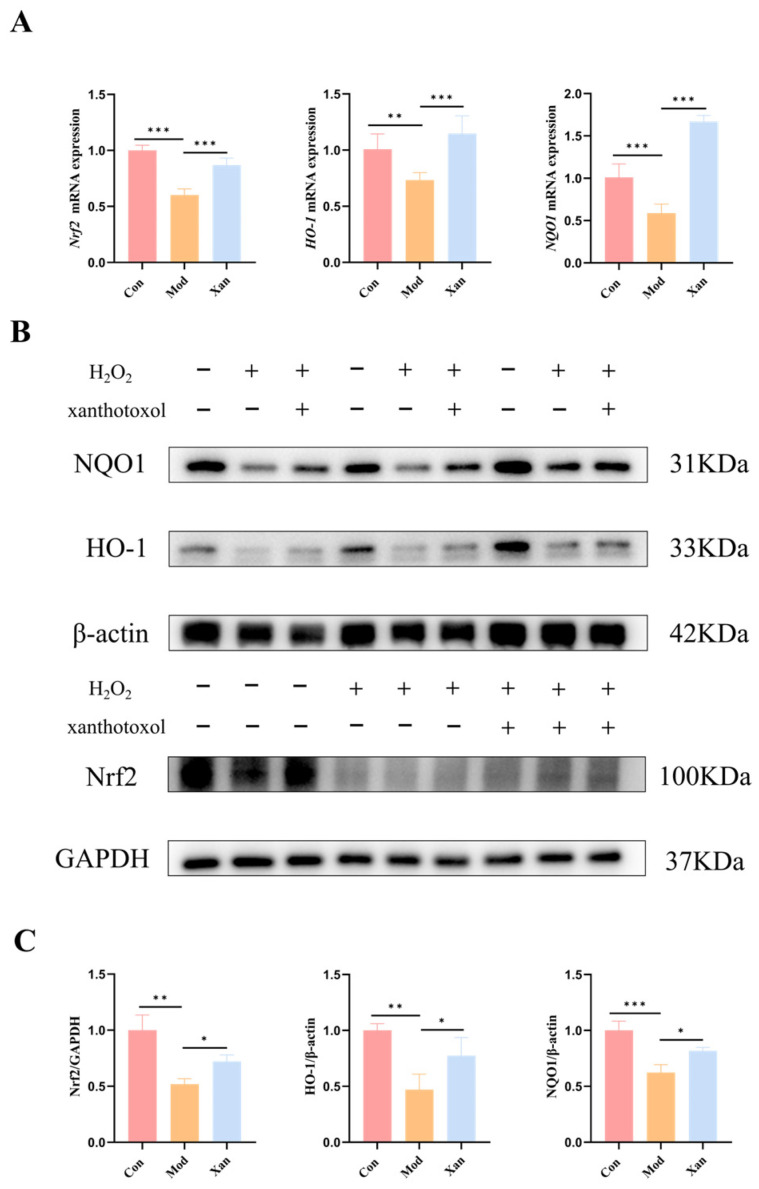
Effect of xanthotoxol on the expression of the Nrf2 pathway. (**A**) *Nrf2*, *HO-1* and *NQO1* mRNA expression. (**B**,**C**) Western blot analysis. “+” means added, “−” means not added. Control group (Con); H_2_O_2_-induced aging model group (Mod); H_2_O_2_-induced cells treated with 8 μmol/L xanthotoxol (Xan). Statistical significance: * *p* < 0.05, ** *p* < 0.01, *** *p* < 0.001.

To elucidate the mechanism underlying the anti-aging effect of xanthotoxol, the expression of genes and proteins associated with the Nrf2 pathway was assessed by means of RT-qPCR and Western blot analysis. Compared with the control group, the mRNA levels of *Nrf2*, *NQO1*, and *HO-1* were significantly downregulated in the model group (*p* < 0.05; Figure 10A). In contrast, the treatment with xanthotoxol significantly elevated the mRNA levels of these genes relative to the model group (*p* < 0.05). Consistently, the protein expression levels of Nrf2, NQO1, and HO-1 were significantly downregulated in the model group compared with the control group (*p* < 0.05; Figure 10B,C), whereas xanthotoxol treatment significantly elevated the expression of all three proteins compared with the model group (*p* < 0.05). These results demonstrated that xanthotoxol activated the Nrf2 signaling pathway, enhanced antioxidant capacity, reduced oxidative damage, and thereby contributed to delaying the aging process.

## 4. Discussion

*Vaccinium bracteatum* Thunb. leaves (VBTL), a plant with a millennial history of culinary and medicinal use in China, represents a bioactive-rich dietary component. Although its in vitro antioxidant capacity has been documented, its in vivo anti-aging efficacy and underlying mechanisms remain unknown. This study provides integrated evidence that VBTL extracts exert potent anti-aging effects across species, primarily via activation of the Nrf2 pathway, with the coumarin derivative xanthotoxol identified as a pivotal constituent. Our results provide critical evidence supporting the development of VBTL into a functional food ingredient or anti-aging nutraceutical.

VBTL extracts demonstrated consistent and superior anti-aging efficacy across all experimental models. In the D-galactose-induced mouse model, VBTL extracts effectively alleviated oxidative stress and tissue damage. Specifically, VBTL treatment restored the activities of key antioxidant enzymes (SOD, CAT, GSH-Px) in the liver, reduced MDA levels, and improved liver histopathology (Figure 2). These protective effects are consistent with those reported for other plant-based interventions such as raisin polyphenols [39] and *Stevia* residue extract [40], supporting a common mechanism of enhancing endogenous antioxidant defenses. Notably, a comparative analysis with *C. unilocularis* shoot extract [41] revealed a distinct potency advantage of VBTL extracts. Although both extracts mitigated oxidative stress markers, administration of VBTL at 400 mg/kg restored the activities of SOD, CAT, and GSH-Px in the liver by 66.46%, 52.15%, and 23.72%, respectively, and reduced MDA levels by 54.80%. These effects substantially surpassed those achieved by *C. unilocularis* extract even at a higher dose of 600 mg/kg, which elevated the same enzymes by 24.98%, 31.44%, and 17.68% and decreased MDA by 23.38%. This superior efficacy was also observed in *C. elegans*, where VBTL not only enhanced antioxidant enzymes and reduced lipofuscin but also extended lifespan by 18% at 1 mg/mL—an effect surpassing reports for other dietary extracts like *Hypsizygus marmoreus* [42], mulberry leaf [43] and Tsai Tai [44]. *Hypsizygus marmoreus* extract at 5 mg/mL extended lifespan by approximately 3.46%, mulberry leaf extract (1 mg/mL) by 6.36%, and Tsai Tai extract (2 mg/mL) by 8%. The superior efficacy may be attributed to a richer or more synergistic bioactive profile in VBTL, leading to more robust activation of endogenous defense systems. A key finding of this study is that VBTL possesses a unique phytochemical profile compared to other species within the genus *Vaccinium*. Within the *Vaccinium* genus, existing studies have predominantly focused on fruit extracts, and their anti-aging effects have largely been attributed to anthocyanins. For example, *Vaccinium uliginosum* extract, owing to its high anthocyanin content, ameliorates UVB-induced skin photoaging [45]. Bilberry (*Vaccinium myrtillus* L.), which is also rich in anthocyanins, not only alleviates aging-induced oxidative stress but also prolongs the average and maximum lifespans of *Drosophila melanogaster*, and enhances their reproductive capacity as well as the activity of antioxidant enzymes [46,47]. In contrast, metabolomic analysis in this study revealed that the linear furanocoumarin xanthotoxol is one of the potential key bioactive components responsible for the effects of VBTL extracts.

In H_2_O_2_-induced cellular senescence, xanthotoxol treatment significantly mitigated cytotoxicity, reduced ROS and SA-β-gal positivity, enhanced SOD activity, and lowered MDA levels. These results corroborate the findings of studies on other natural products, such as *Lycium ruthenicum* Murray anthocyanins [48]. Furthermore, xanthotoxol downregulated the expression of core SASP factors (IL-6, MMP1, MMP3) [49] and key cell-cycle arrest mediators (*P53*/*P21*/*P16*) [50,51,52]. This aligns with prior reports, such as the effect of puerarin on SASP and aging markers in vivo [53], indicating that the action of xanthotoxol extends beyond merely alleviating oxidative stress to modulating fundamental senescence pathways.

A central mechanistic insight from this study is the activation of the Nrf2/Keap1 pathway by both VBTL extracts and xanthotoxol. We found that D-gal or H_2_O_2_ treatment suppressed the expression of Nrf2 and its downstream effectors HO-1 and NQO1. This suppression occurred at both the gene and protein levels (Figure 4 and Figure 10), which was effectively reversed by VBTL or xanthotoxol intervention. This observation aligns with a growing body of evidence identifying Nrf2 as a key molecular target for anti-aging interventions. Previous studies have demonstrated that other natural products like E Se tea extract [54] and curcumin [55] also exert protection via Nrf2 activation. Similarly, resveratrol [56] has been shown to activate the Nrf2 signaling pathway, enhance the activity of antioxidant enzymes such as SOD and CAT, and improve cognitive function as well as lifespan. Sulforaphane [57], one of the most potent natural Nrf2 activators, effectively inhibits NF-κB-mediated inflammatory processes and alleviates inflammaging. Molecular docking simulations predicted a stable interaction between xanthotoxol and key residues in the binding pocket of the Keap1 protein, with a favorable affinity (−7.9 kcal/mol). This computational evidence suggests a plausible mechanism whereby xanthotoxol competitively inhibits Keap1-mediated ubiquitination of Nrf2, thereby promoting Nrf2 stabilization and nuclear translocation.

This study provides comprehensive evidence, utilizing multiple models including D-galactose-induced aging mice, *C. elegans*, and H_2_O_2_-induced cellular senescence, demonstrating that VBTL represents a promising source of anti-aging bioactives. Experimental results confirmed that VBTL effectively alleviates oxidative stress, enhances the activity of endogenous antioxidant enzymes, and significantly extends the lifespan of model organisms. Through untargeted metabolomics combined with molecular docking analysis, followed by validation with in vitro cellular experiments, xanthotoxol was identified as one of the key bioactive constituents mediating these effects. However, this study is limited by its reliance on exogenous stress-induced aging models, which may not fully recapitulate natural aging. Furthermore, while xanthotoxol is a key component, potential synergistic interactions within the complex VBTL matrix warrant further investigation.

## 5. Conclusions

In summary, our findings demonstrate that VBTL extracts, with xanthotoxol as an active constituent, significantly alleviate oxidative stress and aging induced by D-galactose and H_2_O_2_. The underlying mechanism appears to involve the activation of the Nrf2 signaling pathway, thereby enhancing the endogenous antioxidant defense system. These findings highlight the potential of VBTL extracts as a natural source of anti-aging agents and support their further development as functional ingredients for the prevention or mitigation of oxidative stress-related aging.

## Figures and Tables

**Table 1:** RT-qPCR primer sequences.

Genes	Forward Primer	Reverse Primer
*Nrf2*	TGCCACCGCCAGGACTACAG	GCGTGCTCAGAAACCTCCTTCC
*NQO1*	CATTGCAGTGGTTTGGGGTG	GAGTACATGGAGCCGCTACC
*HO-1*	AGCCCCACCAAGTTCAAACA	CATCACCTGCAGCTCCTCAA
*GAPDH*	GACTCCACTCACGGCAAATTCAAC	GACACCAGTAGACTCCACGACATAC

**Table 2:** RT-qPCR primer sequences.

Genes	Forward Primer	Reverse Primer
*IL6*	AGAGTAGTGAGGAACAAGCCAGAG	GGCATTTGTGGTTGGGTCAGG
*MMP1*	TACGAATTTGCCGACAGAGATGAAG	ACAGTTCTAGGGAAGCCAAAGGAG
*MMP3*	TGATGATGATGAACAATGGACAAAGG	AGGTCTGTGAGTGAGTGATAGAGTG
*P53*	CCTCTCCCCAGCCAAAGAAG	CTTCAGGTGGCTGGAGTGAG
*P21*	GCCCGTGAGCGATGGAACTTC	CCTGCCTCCTCCCAACTCATCC
*P16*	CCGTGGACCTGGCTGAGGAG	CGGGGATGTCTGAGGGACCTTC
*Nrf2*	GGTTCCAAGTCCAGAAGCCA	GGTTGGGGTCTTCTGTGGAG
*NQO1*	GGCTTCCAAGTCTTAGAACCTCAAC	TCTCCAGGCGTTTCTTCCATCC
*HO-1*	GCCAGTGCCACCAAGTTCAAG	GATGTTGAGCAGGAACGCAGTC
*GAPDH*	GTCAAGGCTGAGAACGGGAA	AAATGAGCCCCAGCCTTCTC

## Data Availability

The original contributions presented in this study are included in the article. Further inquiries can be directed to the corresponding authors.

## Abbreviations

BCA	bicinchoninic acid
CAT	catalase
*C. elegans*	*Caenorhabditis elegans*
Gal	D-galactose
DCFH-DA	2′,7′-Dichlorodihydrofluorescein diacetate
DMEM	Dulbecco’s Modified Eagle Medium
GSH-Px	glutathione peroxidase
HO-1	heme oxygenase-1
IL-6	interleukin-6
Keap1	Kelch-like ECH-associated protein 1
KNN	k-nearest neighbors
MDA	malondialdehyde
MMP1	matrix metalloproteinase-1
MMP3	matrix metalloproteinase-3
NGM	nematode growth medium
NQO1	NAD(P)H quinone oxidoreductase 1
Nrf2	nuclear factor erythroid 2-related factor 2
PBS	phosphate-buffered saline
qRT-PCR	quantitative real-time polymerase chain reaction
ROS	reactive oxygen species
SOD	superoxide dismutase
SA-β-gal	senescence-associated β-galactosidase
SASP	senescence-associated secretory phenotype
SVR	support vector regression
VBTL	*Vaccinium bracteatum* Thunb. leaves
Vc	vitamin C
Xan	xanthotoxol
(PE P−18:1/22:6)	1−_1Z−Octadecenyl_−2−_4Z_7Z_10Z_13Z_16Z_19Z−docosahexaenoyl_−sn−glycero−3−phosphoethanolamine
PE(22:6/22:6)	PE_22_6_4Z_7Z_10Z_13Z_16Z_19Z_/22_6_4Z_7Z_10Z_13Z_16Z_19Z__
THC−SO_4_H	{5−[2_3−dioxo−3−_2_4_6−trihydroxyphenyl_propyl]−2−hydroxyphenyl}oxidanesulfonic acid
TAG 22:6/20:0/14:0	[_2S_−2−icosanoyloxy−3−tetradecanoyloxypropyl]_4Z_7Z_10Z_13Z_16Z_19Z_−docosa−4_7_10_13_16_19−hexaenoate
DMC−Chal−OSO_3_H	{4−[3−_5−hydroxy−2_2−dimethyl−2H−chromen−6−yl_prop−2−enoyl]phenyl}oxidanesulfonic acid
Rapamycin	_1R_12S_16Z_24E_26E_28Z_32S_−1_18−dihydroxy−12−[1−_4−hydroxy−3−methoxycyclohexyl_propan−2−yl]−19_30−dimethoxy−15_17_21_23_29_35−hexamethyl−11_36−dioxa−4−azatricyclo[30.3.1.0_]hexatriaconta−16_24_26_28−tetraene−2_3_10_14_20−pentone
